# Wind loss model for the thick canopies of orchard trees based on accurate variable spraying

**DOI:** 10.3389/fpls.2022.1010540

**Published:** 2022-09-23

**Authors:** Chenchen Gu, Wei Zou, Xiu Wang, Liping Chen, Changyuan Zhai

**Affiliations:** ^1^Intelligent Equipment Research Center, Beijing Academy of Agriculture and Forestry Sciences, Beijing, China; ^2^National Engineering Research Center for Intelligent Equipment in Agriculture, Beijing, China; ^3^National Engineering Research Center for Information Technology in Agriculture, Beijing, China

**Keywords:** wind variable application, wind loss, regression algorithm, canopy thickness, leaf area, LiDAR

## Abstract

Variable application by wind is an efficient application technology recommended by the Food and Agriculture Organization (FAO) of the United Nations that can effectively improve the deposition effect of liquid medicine in a canopy and reduce droplet drift. In view of the difficulty of modelling wind forces in orchard tree canopies and the lack of a wind control model, the wind loss model for a canopy was studied. First, a three-dimensional wind measurement test platform was built for an orchard tree canopy. The orchard tree was located in three-dimensional space, and the inner leaf areas of the orchard tree canopy and the wind force in different areas were measured. Second, light detection and ranging (LiDAR) point cloud data of the orchard tree canopy were obtained by LiDAR scanning. Finally, classic regression, partial least squares regression (PLSR), and back propagation (BP) neural network algorithms were used to build wind loss models in the canopy. The research showed that the BP neural network algorithm can significantly improve the fitting accuracy of the model. Under different fan speeds of 1,381 r/min, 1,502 r/min, and 1,676 r/min, the coefficient of determination (R^2^) of the model were 81.78, 72.85, and 69.20%, respectively, which were 19.38, 7.55, and 12.3% higher than those of the PLSR algorithm and 21.48, 22.25, and 24.3% higher than those of multiple regression analysis. The comparison showed that the BP neural network algorithm obtains the highest model accuracy, but because the model is not intuitive, PLSR has the advantages of intuitive and simple models in the three algorithms. In practical applications, the wind loss model based on a BP neural network or PLSR can be selected according to the operational requirements and software and hardware conditions. This study can provide a basis for wind control in precise variable spraying and promote the development of wind control technologies.

## Introduction

Pesticide spraying can effectively control diseases and pests and improve fruit quality and fruit yield (Eugen et al., 2017; Nuyttens et al., 2017; Gu et al., 2020). At present, continuous and undifferentiated application is widely used for pesticide applications in orchards, which presents a series of economic and ecological problems such as large amounts of pesticide spraying, low utilization rates, excessive pesticide residues in agricultural products, and environmental pollution (Abbas et al., 2020; Manandhar et al., 2020; Song et al., 2020). Accurate variable spraying can be utilized according to information on crop canopy characteristics, realizing pesticide application according to the presence or absence of crops, canopy volume, and density and effectively addressing problems in existing application operations (Zhou et al., 2017; Colaço et al., 2018), which can effectively promote sustainable agricultural economy and ecology.

Variable wind applications can disturb leaves, enhance the ability of droplets to penetrate and deposit in the canopy, and improve spray operation quality (Gu et al., 2014). This approach is an internationally recognized technical means to effectively improve the utilization rate of pesticides (Chen et al., 2017; Li et al., 2017). At present, research on wind regulation is in the primary stages of development, the regulation method is not mature, and there is a lack of effective control models. Whether wind control is appropriate during the application process directly affects the operational effect (Al-Jumaili and Salyani, 2014; Chen et al., 2017; Song et al., 2017). If the wind force is too small, the chemical solution cannot penetrate the surface of the canopy and deposit inside the canopy, resulting in incomplete disease prevention and control, increasing the occurrence of diseases. Excessive wind force will cause the drifting of liquid medicine, polluting the soil and the surrounding environment and endangering humans and livestock. Khot et al. (2012) studied variable spraying under different wind conditions and walking speeds. Their test results showed that a 70% air-assisted spray was more effective than the 100% air-assisted spray, which can effectively reduce droplet drift. Wind regulation methods have mainly focused on wind regulation technologies and sprayer devices, the establishment of distribution methods for wind and fog fields outside a canopy, and so on. There is less research on the distribution of wind fields and wind demand and loss models in orchard tree canopies (Zhai et al., 2018). Accurately regulating the wind force of a spray and studying the influence law of wind force on fog droplets in a canopy is the focus of this current research.

Wind regulation includes wind direction, wind speed, and wind volume. The basis of wind regulation is to control the direction of air supply consistent with the direction of spray (Duga et al., 2015b). Wind speed and wind volume are the main research topics of wind regulation, and the two are coupled relationships. Through the coordinated regulation of the air inlet and outlet of a sprayer fan, wind force regulation can be achieved to provide appropriate wind force for an orchard tree canopy and ensure that the liquid medicine evenly covers the fronts and backs of leaves and the surfaces of orchard tree branches. The distribution of wind power in space and the canopy of an air-delivered sprayer is mainly studied through computational fluid dynamics (CFD) simulation technology. CFD technology can reduce the cost of wind prediction, and it is an important means to study the wind fields of sprayers. Dekeyser et al. (2013) used CFD to simulate a wind field outside the air outlet of a horizontal axis sprayer, distribution sprayer, and independent nozzle air sprayer. Through experimental verification, it was concluded that the CFD simulated wind field can better fit the experimental data. Duga et al. (2015a) studied the influence of external wind force and spray type on spray distribution in different orchards by establishing three-dimensional numerical models of tree crowns for four orchard trees using CFD modeling and orchard test verification methods and improved the quantitative understanding of spray design, wind force, and canopy structure interaction. In the above research, through CFD modeling, the wind field distribution of the sprayer was found to be mostly the wind field outside the canopy. There has been less research on wind fields inside a canopy. Hong et al. (2017) used CDF technology for modeling by using virtual porous media instead of actual trees. It was found that canopy size and canopy density have a great impact on air entering the canopy, and the air velocity will decrease with increasing canopy thickness, tree height, and canopy density. They established a CFD model for the distribution of wind forces in a canopy but did not obtain an effective mathematical model that could directly calculate the wind force in a canopy.

The wind regulation model is the basis of wind regulation. The wind force is affected by the canopy thickness and canopy density during canopy penetration. Most of the existing studies have focused on modern orchards, and the research objects were characterized by small canopy thicknesses, dense branches and leaves, and uniform distribution. The wind loss model can make the wind force regulation reasonable. Through the wind loss model, the wind loss can be calculated through the canopy information, and then the wind speed served by the sprayer can be known. Reasonable wind for spray can improve the uniformity of pesticide deposition and the efficacy. Research on a wind field and wind loss model in a traditional thick canopy has not been carried out. Due to the large changes in canopy thickness and density in different areas of orchard tree canopies, the distribution of wind forces in different areas of a canopy has the characteristics of large differences in change rules and is difficult to measure and quantify, which hinders the establishment of wind control models.

At present, research on variable wind spraying technology has achieved variable spray volume control and wind direction control, and variable spray technology has been preliminarily achieved. However, there is a lack of research on the law of wind change in orchard tree canopies. To study the law of wind loss under different influencing factors in orchard tree canopies, based on previous canopy volume detection (Gu et al., 2021a) and the fitting model of LiDAR point cloud data and the leaf area (Gu et al., 2021a, 2022). By gridding the tree canopy, the wind was measured outside and inside the canopy. The wind loss model in canopies based on the wind speed at the entrance of the canopies, canopy thickness, and canopy leaf area/LiDAR point cloud data are evaluated in this study. The classic regression, PLSR, and BP neural network algorithms are used to establish the wind loss model in canopies, which provides an effective basis for wind regulation for accurate variable spraying and plays an important role in achieving pesticide reduction and increased efficiency.

## Materials and methods

### Test platform

**Figure 1:**
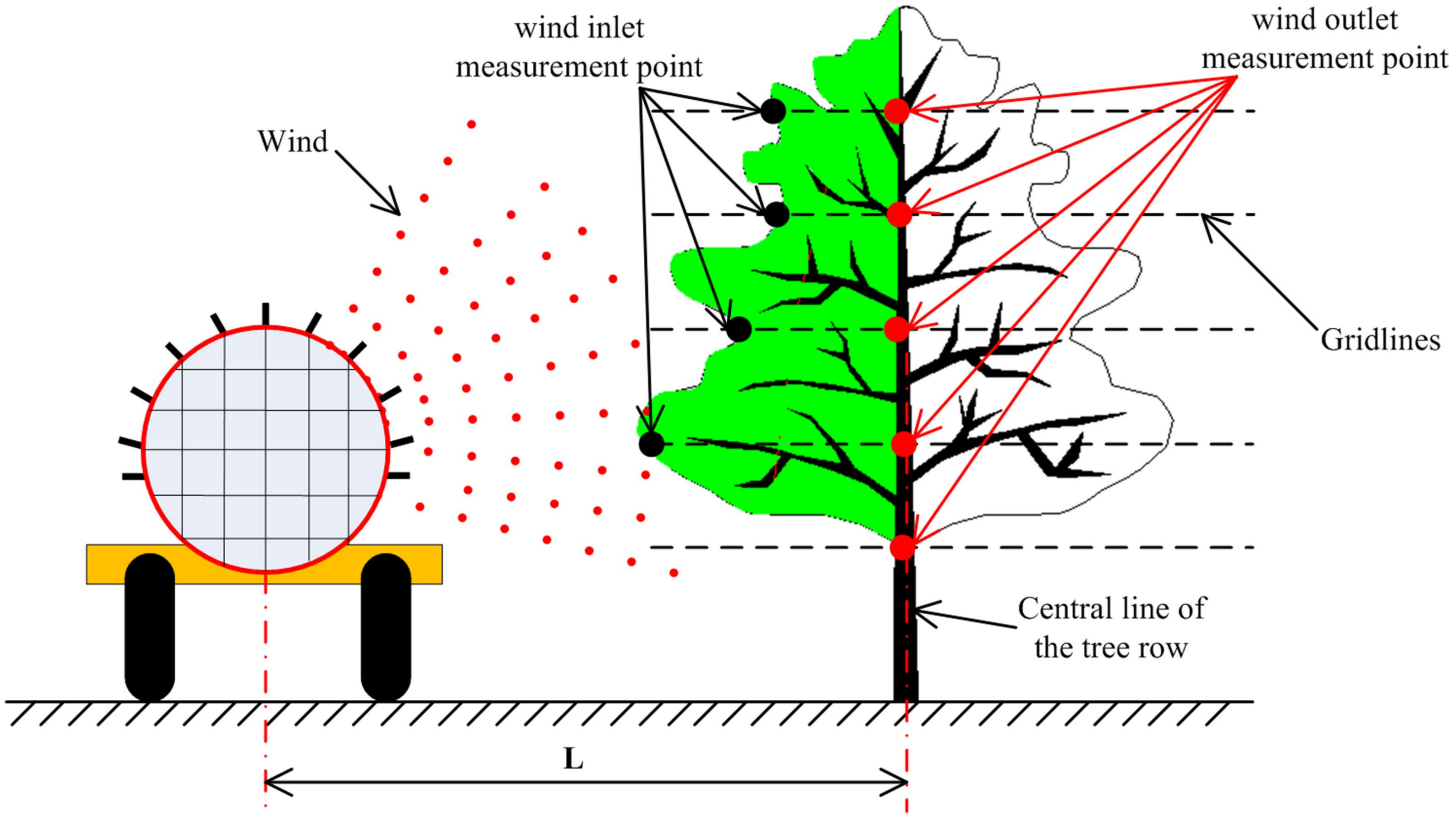
Schematic diagram of the canopy wind measurement.

We designed a three-dimensional measurement platform of orchard tree canopies, enveloped a whole orchard tree canopy in the platform, and achieved the positioning of canopies in three-dimensional space. Figure 1 shows a schematic diagram of the wind measurement process in a canopy. The sprayer sends air through the air supply system. The sprayer applies the pesticide on one side of the orchard tree when working between rows, and then spray on the other side when the row changed. According to the operation mode of the sprayer, only one side of the orchard tree canopy was studied. In the wind measurement of canopy, the outermost side of the tree is the wind inlet measurement point, and the central line of the tree row is the wind outlet measurement point. The wind force at the canopy inlet (black measurement point) and outlet (red measurement point) is measured by a wind meter, and L is the distance (2 M) from the wind supply center of the sprayer to the position of the orchard tree row during the wind measurement process. A Langshan 3 WGF-300D air-driven orchard applicator is used for air supply. The working pressure of the applicator is 1.2–1.5 MPa, the flow of the medicine pump is 60 l/min, the fan speed is 0–2,800 r/min, the volume of the medicine box is 300 l, and the overall dimension is 2.5 × 1.3 × 1.16 M. Moreover, the spray width is greater than or equal to 20 M, the spray height is greater than or equal to 7 m, and the operation speed is 3–4.2 km/h. The thermal anemometer is used to measure the wind force in the canopy. The model is gm8903, the measurement range is 0–30 m/s, and the resolution is 0.001 m/s. The canopy between the measurement points of the canopy inlet and canopy outlet is the wind measurement canopy area (green area).

**Figure 2:**
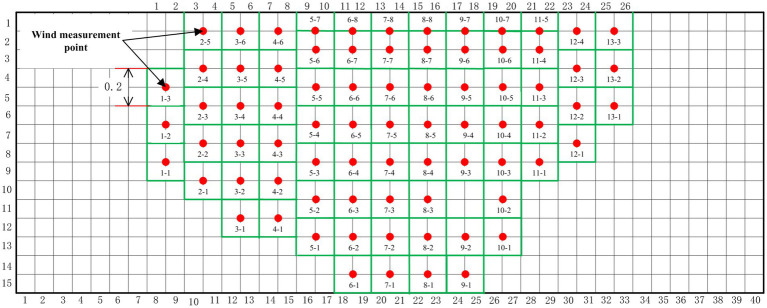
Canopy grid division and measurement point marking for the airflow test.

According to the distribution of the orchard tree canopy in the grid area, the canopy is divided into different measurement areas by dividing lines. The number of measurement areas is the same as the number of applicator nozzles, and the measurement area is divided into 0.2 × 0.2 M (Figure 2), realizing the positioning of different measurement areas in the canopy area. Marking the positions of wind measurement points at the entrance and exit of the canopy with label paper is conducive to the smooth progress of wind speed measurement in the canopy. In Figure 2, the red point is the measurement position mark point, and the green frame is the measurement division area.

### Test orchard tree

**Figure 3:**
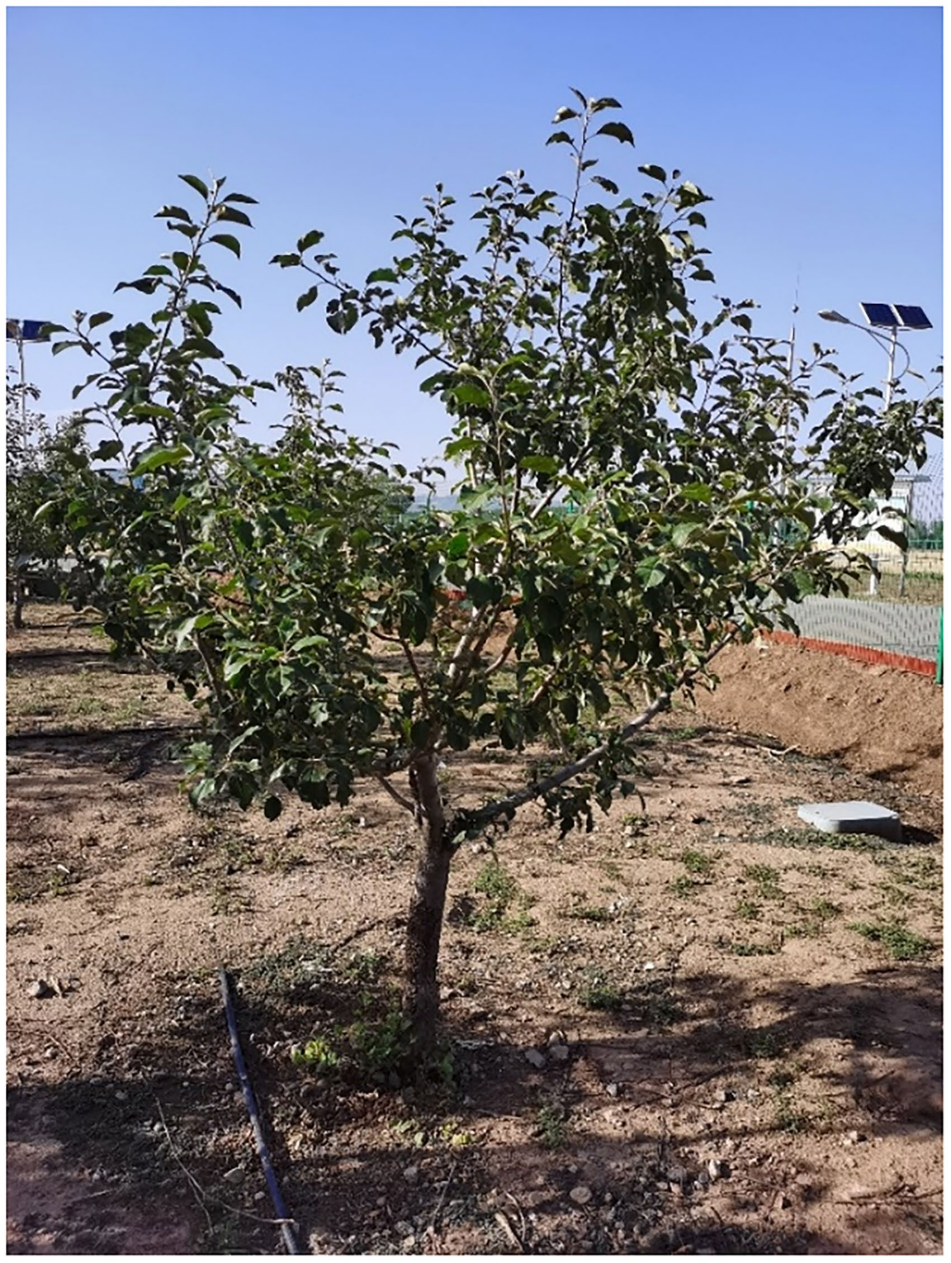
Test orchard tree.

The selected orchard tree in the test is shown in Figure 3. The test site is the Xiaotangshan National Precision Agriculture Research Demonstration Base in Changping District, Beijing. The test tree was one Fuji apple tree, and the tree was 5 years old. The tree used in the research is open center tree shape (Gao et al., 2015). It has the advantages of obvious middle trunk, main branch, and natural stratification. During the processes of growth, the tree pruning amount is light, growth and formation are fast, and bearing of fruits early. The height of the tree was 2.3 M, the lower edge of the canopy was 0.8 M from the ground, and the crown was 1.5 M high and 2.5 M wide. The row spacing is 4 M and the plant spacing is 3.5 M. The experimental research time was October 11, 2020. According to the definition of the growth stage of mono- and dicotyledonous plants (Bleiholder et al., 2001), the apple tree is in the final stage of the principal growth stage: maturity of the fruit and seed; fruit ripening for consumption; and fruit achieving typical taste and firmness. At this time, the apples are mature with typical taste and hardness. Apple trees in the orchard have no fallen leaves, and the distribution of the leaf area in the canopy has not changed.

### Test method of wind in the canopy and natural wind measurement

Before the test, the air supply width of different wind forces of the sprayer was determined, the measurement position was set to 1, 1.5, and 2 M away from the fan outlet, and the air supply width range of the wind force from the sprayer outlet to the measurement position was measured based on a position 1 M away from the horizontal ground in the vertical direction. During the measurement process, different applicator fan speeds were set, the wind speed boundary was measured as 2 m/s, the horizontal displacement between the measurement position and the left and right sides of the air outlet was recorded, and the wind supply width of the sprayer was determined.

**Figure 4:**
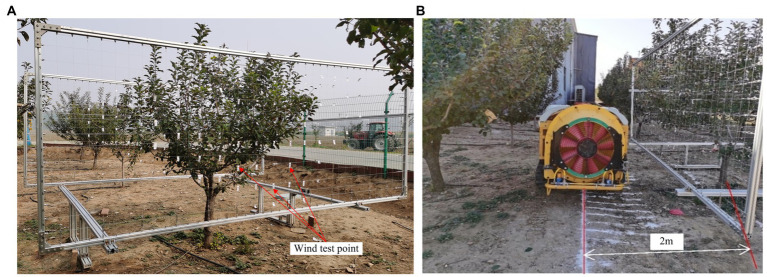
Canopy airflow measurement test. **(A)** Layout of canopy airflow test points. **(B)** Canopy airflow measurement test.

The wind measurement points were laid out on a three-dimensional measurement test bench, as shown in Figure 2, and the wind speed measurement points were marked at the entrance and exit of the canopy with label paper (Figure 4A). The air supply width was determined according to the results. Before the test, the moving distance of the applicator was calibrated. After each measurement, the wind speed at the corresponding canopy position at the air outlet of a group of sprayers moved forward and continued to measure the next test unit, as shown in Figure 4B. During the experiment, the sprayer is in the middle of tree row. The center of sprayer from the center line of tree row is 2 M, and consistent with the distance of the fruit farmers’ planting operation.

**Figure 5:**
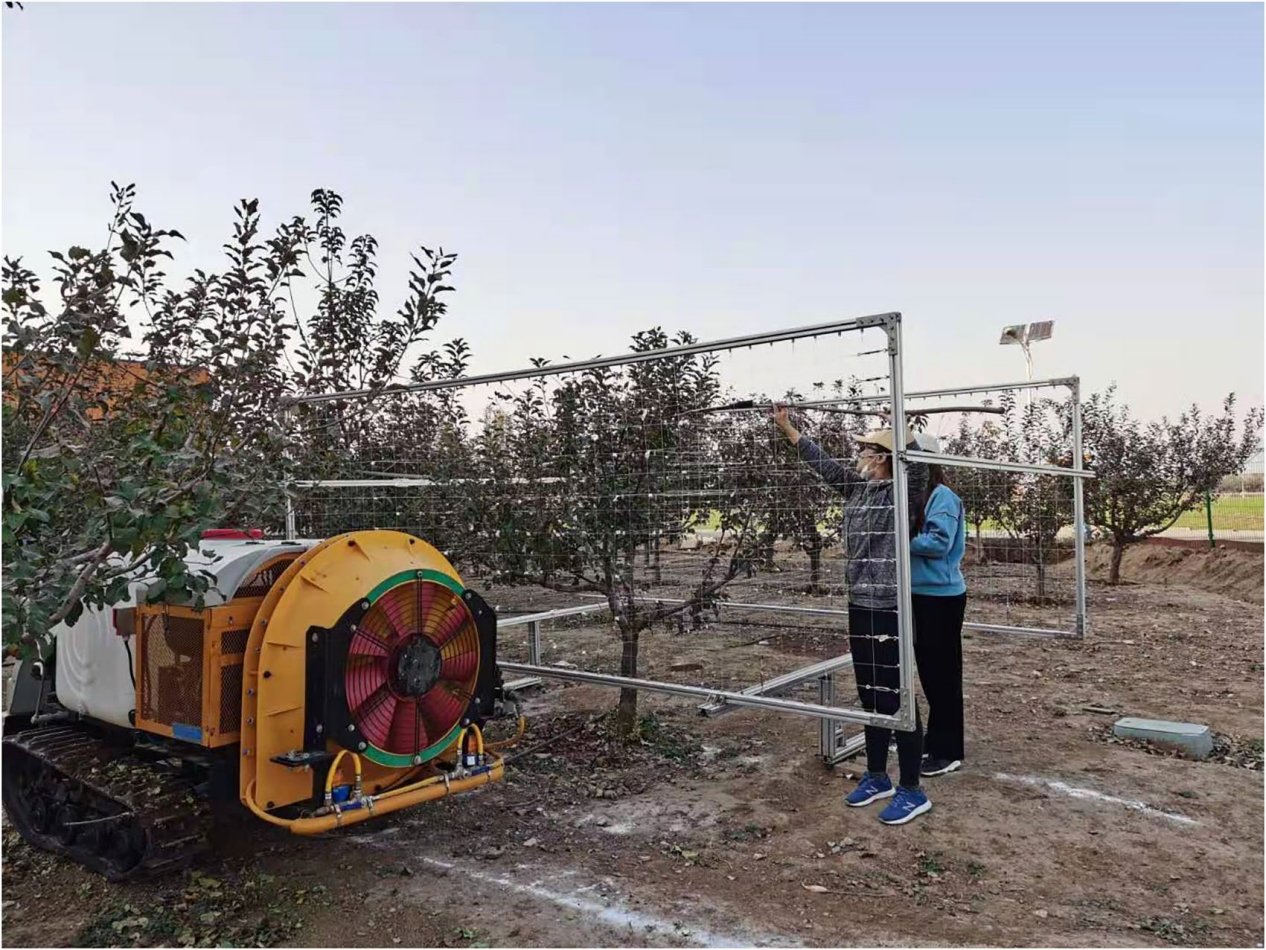
Airflow measurement in the canopy.

During the measurement of the wind speed at the wind inlet and outlet at the marked position of the canopy, to detect the wind force at different positions in the canopy and reduce artificial interference, an anemometer measuring probe was fixed on the 1.5 M probe rod (Figure 5), the probe was placed at the measuring point in the canopy through the probe rod, the wind speed was measured, and the wind value at each measuring position was read 3 times.

When the orchard experiment was conducted, the nature wind speed was measured. We set up a WindSonic portable weather station which was used to measure natural wind speed in the orchard. The data was obtained once per minute.

### Data processing method of the wind measurement test in the canopy

During the test, the fan speeds of the sprayer were set to 1,381, 1,502, and 1,676 r/min, and the wind speeds at the inlet and outlet of the wind were obtained to conduct an experimental study on the wind loss in the canopy. The three levels of spray fan speed represent different air-supplied conditions for the diversity of test conditions. The wind loss model is different with different air supply speed. The canopy wind loss rate was calculated using the inlet and outlet wind speeds of the canopy wind, and the calculation formula is:


(1) $$\begin{array}{l} \mathrm{SpeedLos}s_{C\mathrm{anopy}}=\\ \left(\mathrm{Spee}d_{C\mathrm{anopyIN}}-\mathrm{Spee}d_{C\mathrm{anopyOUT}}\right)/\mathrm{Spee}d_{C\mathrm{anopyIN}} \end{array}$$

where,

*SpeedLoss_Canopy_* - wind speed loss rate.

*Speed_CanopyIN_* - inlet wind speed, m/s.

*Speed_CanopyOUT_* - outlet wind speed, m/s.

Due to the influence of the location of air inlets and outlets and the external natural wind, any unreasonable data groups need to be removed in the process of calculating the wind loss. First, according to the wind blowing process, the wind speed at the canopy outlet should be able to continuously disturb the leaves (Dai, 2008), ensure that the liquid medicine is evenly deposited on the fronts and backs of the leaves at the canopy outlet, and remove the measurement points where the wind speed at the canopy wind outlet is zero. Due to the influence of natural wind, the measurement points where the wind speed at the canopy inlet is less than that at the canopy outlet are removed. Due to the small canopy thickness and density of branches and leaves at individual positions of the canopy, the data of measurement points with equal wind speed at the entrance and exit of the canopy are removed. Using the above process, effective data for the study of the wind loss model of the orchard tree canopy are obtained.

### Measurement of canopy thickness and leaf area

Before the wind measurement test, the canopy thickness and leaf area of different areas of the orchard tree canopy were measured. The canopy thickness measurement adopted the canopy volume detection method CMC (canopy meshing profile characterization; Gu et al., 2021b) obtained in previous research to calculate the thickness of different canopy positions.

**Figure 6:**
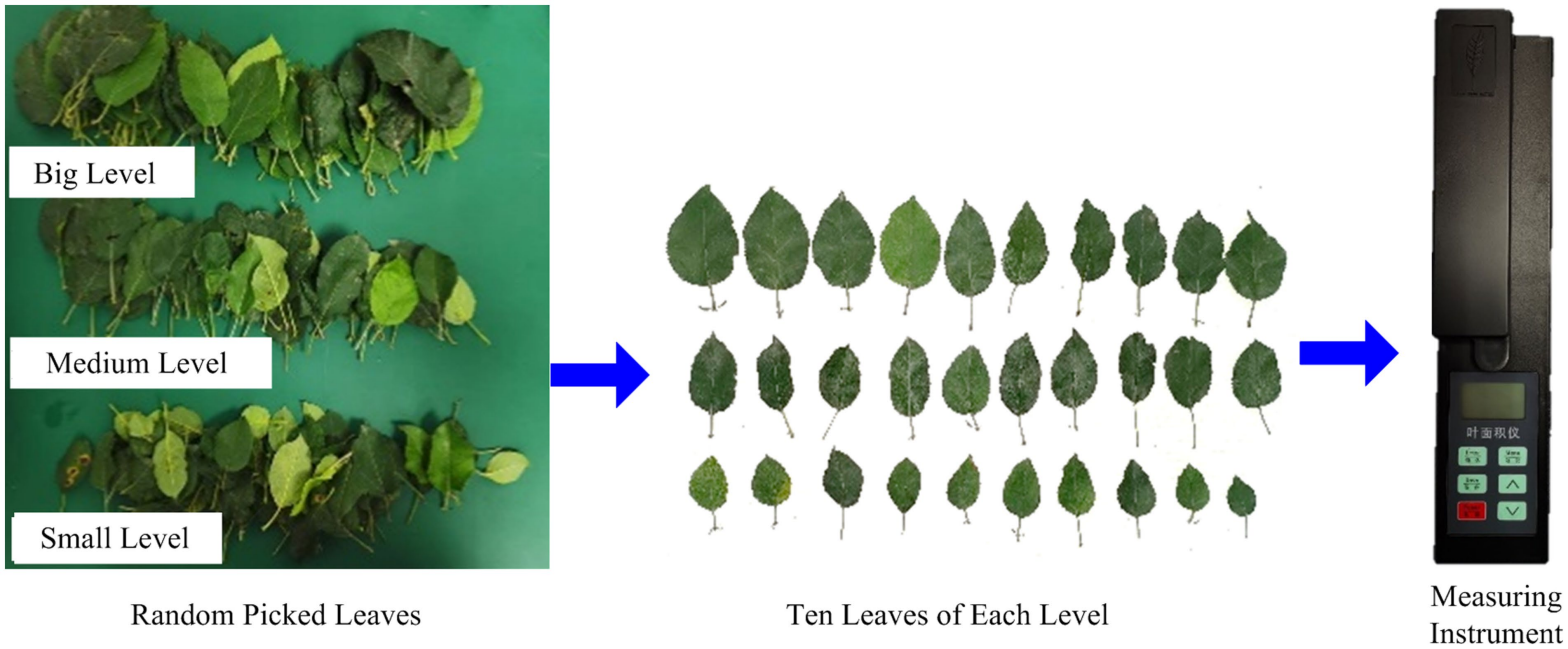
Leaf area measurement by statistical analysis.

The measurement method for the canopy leaf area adopts a non-destructive statistical measurement method to calculate the leaf area for different areas in the canopy (Figure 6). To manually measure the leaf area of apple trees in different areas, a three-dimensional measurement grid frame of canopy leaf area is used to divide the apple tree canopy into different areas. It is necessary to count the number of leaves in the three grades of large, medium, and small leaves in the measurement area, multiply the calculated average value of leaf area in each grade by the number of leaves in each grade, and sum the leaf area calculated in each grade to obtain the sum of the leaf area in this area. The leaf area obtained by statistical analysis was compared with the total leaf area measured by the leaf area instrument one by one, and the relative error was 1.8% (Gu et al., 2022). The accuracy is high and is feasible and appropriate for this experimental study.

### Wind loss model

To obtain the wind speed at the entrance of the canopy, the wind stroke in the canopy, the leaf area/LiDAR point cloud data in different areas of the canopy, and the wind loss rate in the canopy under different wind conditions, multiple regression, PLSR, and BP neural network algorithms that can perform regression analysis on multiple dependent variables and multiple independent variables were used.

Classic regression analysis was carried out using Minitab software; the appropriate relationship model was selected, the regression statistics were stored, the residual analysis and confidence interval were tested, and a lack-fit test was carried out. When creating regression equations, the PLSR algorithm considers extracting the principal components of dependent variables and independent variables (principal component analysis: PCA) and extracting the maximum correlation between principal components (canonical correlation analysis: CCA). This is the product of three basic algorithms, PCA, CCA, and multiple linear regression, which can remove the redundancies among data to the greatest extent. The BP neural network regression algorithm can carry out more accurate regression on multiple influencing factors and obtain an accurate model. 77 sets of data were obtained in the research. To prevent the obtained model from fitting the training set well, the fitting effect of data other than the training data is inconsistent. 70% of the data are used for model establishment, and the remaining 30% of the data are used for model verification. The data set was disorganized in the process, and the training and test set was randomly extracted.

## Results

In this study, the selected object is orchard trees that are widely planted in Chinese apple orchards and have large canopy thicknesses. During the experiment, the natural wind speed range was 0.2 m/s-0.9 m/s, less than 1 m/s. Since the air-assisted sprayer supplied high-intensity wind, the interior of the tree canopy would not be affected by the natural wind. The concept of canopy zoning is used to study the wind loss model, which meets the actual requirements of orchard pesticide application.

### The sprayer ranges of the air delivery width

Table 1 shows that there is a large gap between the first group and the second group at the fan speed range of 490 r/min. The reason is that the natural wind force interferes with the wind force measurement resulting in the wind turbine wind force measurement process. At fan speeds of 1,207 and 1,280 r/min, the measured fan speed range is relatively uniform. The measured values in Table 1 indicate that the air supply range of the sprayer can be set to 0.2 M. This is consistent with the wind grid size of 0.2 × 0.2 M set in this study.

**Table 1:** The measurement range of the air delivery width of the sprayer.

Fan speed (r/min)	Measurement position from wind outlet (m)	Wind supply width (m)	
490	1	0.18	0.1
	1.5	0.32	0.09
	2	0.33	0.078
1,207	1	0.23	0.1
	1.5	0.4	0.1
	2	0.21	0.23
1,280	1	0.25	0.12
	1.5	0.2	0.13
	2	0.11	0.14

### Normality test of the residual of the wind measurement experimental data

**Figure 7:**
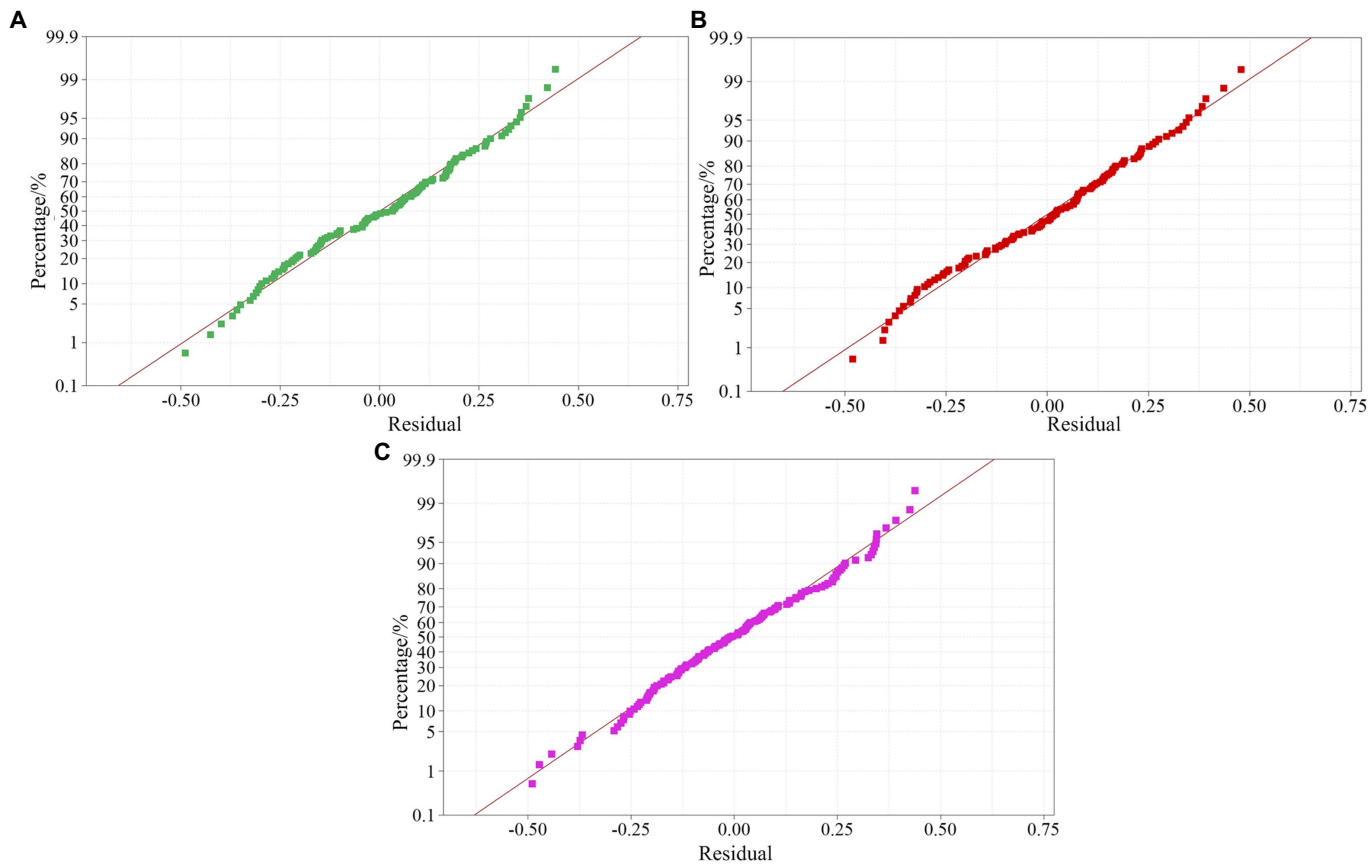
Normal distribution of residuals in the canopy leaf area data set at different sprayer fan speeds. **(A)** 1381 r/min data set. **(B)** 1502 r/min data set. **(C)** 1676 r/min data set.

Before the multiple regression analysis of the data, a residual normal analysis of the test data is required (Figure 7). The normal probability diagram of the residual is approximately a straight line, indicating that the data are randomly distributed, have good fitting to the random error, and can extract all the predictable data ranges.

### Correlation analysis of the research factor interaction items

During the modeling process, in addition to the influencing factors of experimental research, data interaction is key to an accurate model. Table 2 shows a correlation analysis between the two factors of the overall canopy data under different fan speeds. According to Table 2, the correlation between the influencing factors under different fan speeds is generally less than 0.5, which shows that the above factors are independent of each other. During wind loss model research on the data, the interactions between the factors were not considered.

**Table 2:** Correlation analysis of all data before and after the canopy at different speeds.

Fan speed (r/min)	Canopy thickness × inlet wind speed	Canopy thickness × leaf area	Inlet wind speed × leaf area	Canopy thickness × LiDAR point cloud data	Inlet wind speed × LiDAR point cloud data
1,381	0.487	0.416	0.374	0.498	0.490
1,502	0.351	0.400	0.352	0.475	0.541
1,676	0.226	0.412	0.238	0.487	0.404

### Research on wind loss models in canopies based on classic regression algorithms

Based on the canopy leaf area group data and LiDAR point cloud data group, a multiple regression model within the canopy was constructed by using the classic regression method, and the model was evaluated. The canopy inlet wind speed, canopy thickness, canopy leaf area, and canopy wind loss model under different wind conditions were calculated, and the canopy inlet wind speed, canopy thickness, LiDAR point cloud data, and canopy wind loss rate model were evaluated. Formulas 2–7 are the regression models of 1,381 r/min canopy leaf area, 1,381 r/min canopy LiDAR point cloud, 1,502 r/min canopy leaf area, 1,502 r/min canopy LiDAR point cloud, 1,676 r/min canopy leaf area and 1,676 r/min canopy LiDAR point cloud data set in turn:


(2) $$\begin{array}{l} \mathrm{SpeedLos}s_{C\mathrm{anopy}1}=\\ 0.303-1.59\times 10^{-2}x_{1}+0.403x_{2}-1.4\times 10^{-5}x_{3} \end{array}$$

(3) $$\begin{array}{l} \mathrm{SpeedLos}s_{C\mathrm{anopy}2}=\\ 0.301-1.64\times 10^{-2}x_{1}+0.401x_{2}-3\times 10^{-6}x_{4} \end{array}$$

(4) $$\begin{array}{l} \mathrm{SpeedLos}s_{C\mathrm{anopy}3}=\\ 0.341+2.77\times 10^{-2}x_{5}+0.284x_{2}-2.4\times 10^{-5}x_{3} \end{array}$$

(5) $$\begin{array}{l} \mathrm{SpeedLos}s_{C\mathrm{anopy}4}=\\ 0.335+3.16\times 10^{-2}x_{5}+0.286x_{2}-1.8\times 10^{-5}x_{4} \end{array}$$

(6) $$\begin{array}{l} \mathrm{SpeedLos}s_{C\mathrm{anopy}5}=\\ 0.277+1.12\times 10^{-2}x_{6}+0.283x_{2}-9\times 10^{-6}x_{3} \end{array}$$

(7) $$\begin{array}{l} \mathrm{SpeedLos}s_{C\mathrm{anopy}6}=\\ 0.274+1.2\times 10^{-2}x_{6}+0.285x_{2}-6\times 10^{-6}x_{4} \end{array}$$

where:

*SpeedLoss*_Canopy1_—Wind loss rate based on canopy leaf area at 1381 r/min.

*SpeedLoss*_Canopy2_—Wind loss rate based on the LiDAR point cloud of the canopy at 1381 r/min.

*SpeedLoss*_Canopy3_—Wind loss rate based on canopy leaf area at 1502 r/min.

*SpeedLoss*_Canopy4_—Wind loss rate based on the LiDAR point cloud of the canopy at 1502 r/min.

*SpeedLoss*_Canopy5_—Wind loss rate based on canopy leaf area at 1676 r/min.

*SpeedLoss*_Canopy6_—Wind loss rate based on the LiDAR point cloud of the canopy at 1676 r/min.

*x*_1_—wind speed at canopy inlet at 1381 r/min, m/s.

*x*_2_—canopy thickness, m.

*x*_3_—canopy leaf area, cm^2^.

*x*_4_—LiDAR point cloud data of canopy, PCs.

*x*_5_—1,502 r/min, wind speed at canopy inlet, m/s.

*x*_6_—1,676 r/min, wind speed at canopy inlet, m/s.

Table 3 shows the R^2^ of the above regression model (Formulas 3–8), and it is clear that the R^2^ range of the canopy regression model at different speeds is 45–60.4%. The difference between the leaf area and LiDAR point cloud data group in the regression model R^2^ is small, which can be ignored in the range of 0 ~ 0.2%, indicating that the leaf area data and LiDAR point cloud data have the same impact on the wind loss model, and they have a strong correlation. Consistent with the research results of Sanz-Cortiella et al. (2011); Zhang et al. (2017) and Gu et al. (2021a) on the relationship between LiDAR point cloud data and canopy leaf area, when calculating the canopy wind loss model, the leaf area data and LiDAR point cloud data are selected to build the wind loss model. Because LiDAR point cloud data are easier to obtain than the leaf areas of different canopy areas, LiDAR point cloud data are used to replace the canopy leaf area data.

**Table 3:** *R*^2^ values of the regression models under different spray fan speeds.

Project	1,381 r/min		1,502 r/min		1,676 r/min	
	Leaf area	LiDAR	Leaf area	LiDAR	Leaf area	LiDAR
Model	2	3	4	5	6	7
*R* ^2^	60.4%	60.3%	51.2%	51.4%	45%	45%

Table 4 analyses the significance of various test factors on the regression process of the model. In the variance calculation process, the *p* value distribution of LiDAR point cloud data and canopy leaf area in the model is 0.281–0.851, which are both greater than 0.1 and indicate that these two factors have no significant impact on the model. Based on previous research on the distribution of wind fields in a canopy (Hong et al., 2017), it is concluded that density is the main factor affecting the distribution of wind fields in a canopy because the research object is modern orchards, which are characterized by thin canopies and dense leaf distributions. The orchard trees selected in this study are traditional thick canopy orchard trees, and the leaves in the canopy are unevenly distributed, which has little impact on the wind loss under the action of wind. Through the above analysis, the canopy leaf area and LiDAR point cloud data are removed, and the wind loss model of canopy inlet wind speed and overall canopy thickness is further studied. The model of the wind loss rate in the canopy under different rotating speeds of 1,381, 1,502, and 1,676 r/min (Formulas 8–10) are calculated.


(8) $$\mathrm{SpeedLos}s_{C\mathrm{anopy}7}=0.3-1.74\times 10^{-2}x_{1}+0.4x_{2}$$

(9) $$\mathrm{SpeedLos}s_{C\mathrm{anopy}8}=0.335+2.53\times 10^{-2}x_{5}+0.278x_{2}$$

(10) $$\mathrm{SpeedLos}s_{C\mathrm{anopy}9}=0.274+1.07\times 10^{-2}x_{6}+0.281x_{2}$$

where:

**Table 4:** Significance of the factors of the regression models of the canopy wind loss model under different spray fan speeds.

Fan speed r/min	Model	Significance			
		Inlet wind speed	Canopy thick	Leaf area	LiDAR
1,381	2	0.22	0.000	0.604	
	3	0.231	0.000		0.851
1,502	4	0.022	0.000	0.351	
	5	0.017	0.000		0.281
1,676	6	0.267	0.000	0.726	
	7	0.260	0.000		0.721

*SpeedLoss*_Canopy7_—Wind loss rate based on canopy thickness at 1381 r/min.

*SpeedLoss*_Canopy8_—Wind loss rate based on canopy thickness at 1502 r/min.

*SpeedLoss*_Canopy9_—Wind loss rate based on canopy thickness at 1676 r/min.

The R^2^ values of the obtained model are 60.3, 50.6 and 44.9% at 1381 r/min, 1,502 r/min and 1,676 r/min, respectively. Through the study of different canopy wind loss models, it is concluded that the canopy inlet wind speed and canopy thickness are the main influencing factors of wind loss, among which the canopy thickness is more significant. Hong et al. (2017) also demonstrated that canopy thickness is the main factor affecting the distribution of wind in a canopy.

Through the above analysis, it is concluded that the accuracy of the regression model gradually decreases with increasing fan speed because with increasing fan speed, the wind speed at the entrance of the canopy increases, and after the wind enters the canopy, it is affected by factors such as the density and direction of branches in the canopy, resulting in a decline in the fitting effect of the wind loss model. This is consistent with the research results of Khot et al. (2012), who found that 70% wind assistance achieves a better result than 100% wind assistance.

### Research on the wind loss model in a canopy based on the PLSR algorithm

Through classic multiple regression analysis, it is concluded that the correlation coefficient R^2^ of the regression model is small, and the interpretation ability of the prediction data is weak. To obtain a better wind loss model based on multiple regression, the PLSR algorithm is used to study the canopy wind loss model based on canopy inlet wind speed and canopy thickness. Formulas 11–13 are the wind loss models obtained under the conditions of fan speeds of 1,381, 1,502, and 1,676 r/min, respectively.


(11) $$\mathrm{SpeedLos}s_{C\mathrm{anopy}10}=0.282-1.22\times 10^{-2}x_{1}+0.391x_{2}$$

(12) $$\mathrm{SpeedLos}s_{C\mathrm{anopy}11}=0.213+3.39\times 10^{-2}x_{5}+0.319x_{2}$$

(13) $$\mathrm{SpeedLos}s_{C\mathrm{anopy}12}=0.22+1.03\times 10^{-2}x_{6}+0.321x_{2}$$

where,

*SpeedLoss*_Canopy10_—Wind loss rate based on PLSR at 1381 r/min.

*SpeedLoss*_Canopy11_—Wind loss rate based on PLSR at 1502 r/min.

*SpeedLoss*_Canopy12_—Wind loss rate based on PLSR at 1676 r/min.

Table 5 shows that the fitting accuracy of the model obtained by PLSR is higher than that of the multiple regression model by 2.1, 14.7, and 12%. With the increase in the fan speed, the obtained canopy wind loss model R^2^ gradually decreases. As the fan speed increases, the wind speed at the canopy inlet gradually increases, but as the fan outlet of the sprayer remains unchanged, the increase in the wind speed at the canopy inlet does not increase synchronously with the fan speed. The wind loss rate obtained through the wind inlet and the canopy wind outlet fluctuates greatly, resulting in the fitting effect of the model not increasing with increasing speed. At the same time, it is concluded that the prediction of the model for the verification set is weak, and the prediction ability gradually decreases with increasing speed. The reason for this phenomenon is that the wind loss rate gradually increases with the fluctuation of fan speed, and the extraction of verified data sets has a great impact on the verification results. The root mean square error of the data is less than 0.3, indicating a good degree of data concentration.

**Table 5:** *R*^2^ and RMSE of the airflow speed loss rate model under different fan speeds.

Fan speed r/min	Model *R*^2^	Model RMSE	Validation set R^2^	Validation set RMSE
1,381	62.4%	0.262	52.8%	0.164
1,502	65.3%	0.216	33.5%	0.216
1,676	56.9%	0.234	10.8%	0.188

### Study of the wind loss model in the canopy based on the BP neural network

The fitting accuracy of the regression model obtained by the PLSR algorithm is significantly higher than that of the multiple regression model, but the fitting accuracy of the model is still low, at less than 0.7, which cannot predict the canopy wind loss well. The BP neural network can use the error after output to evaluate the leading error of the output layer, update the error of the previous layer, and gradually calculate the errors of other layers to obtain a more accurate regression model calculation of the data. A BP neural network is used to train the wind loss model of the data group at different speeds through the test data group, and the prediction ability of the model to the data is obtained through analysis.

N-fold crossover divides the data set for many times, and averages the results of multiple evaluations, so as to eliminate the adverse effects caused by unbalanced data division in a single division. The model with the best generalization ability can be selected from a variety of models. It can effectively solve the over fitting of data, avoid the limitations and particularity of fixed divided data sets, and have more obvious advantages in small-scale data sets. In the study, 3-fold cross validation and 5-fold cross validation were selected according to the number of data sets (77 sets). The BP neural network-n-fold cross validation method is adopted, and the training sets and test sets with different ratios are used for multiple tests, and the results are averaged.

**Figure 8:**
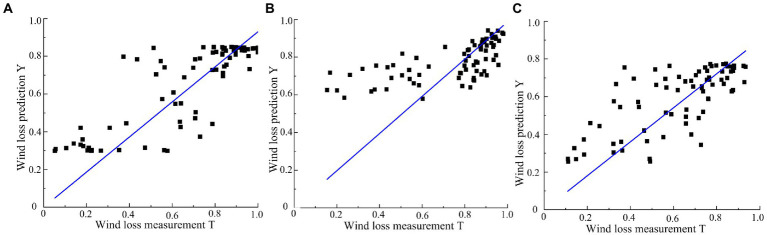
BP neural network training model measurement accuracy based on different conditions. **(A)** 1381 r/min. **(B)** 1502 r/min. **(C)** 1671 r/min.

Under the conditions of different fan speeds of 1,381, 1,502 and 1,676 r/min, the R^2^ of wind loss model of the BP neural network-3-fold cross validation is 76.55, 63.53 and 60.22%. The R^2^ of the BP neural network-5-fold cross validation is 81.78, 72.85 and 69.20%. The models of BP neural network-5-fold cross validation were better than the BP neural network-3-fold cross validation. The accuracy of both the models is higher than that of the model obtained by the PLSR algorithm. The BP neural network-5-fold cross validation are used for the wind loss model. Figure 8 shows the relationship of BP neural network-5-fold cross validation between the predicted value and measured value of wind loss through the trained BP neural network model.

The equation of the two fitting formulas is shown in Formulas 14–16:


(14) $$Y_{1}=0.93T_{1}$$

(15) $$Y_{2}=0.99T_{2}$$

(16) $$Y_{3}=0.90T_{3}$$

where,

*Y*_1_—Prediction value of the wind loss rate of the BP neural network model at 1381 r/min.

*Y*_2_—Prediction value of the wind loss rate of the BP neural network model at 1502 r/min.

*Y*_3_—Prediction value of the wind loss rate of the BP neural network model at 1676 r/min.

*T*_1_—Measured value of wind loss at 1381 r/min.

*T*_2_—Measured value of wind loss at 1502 r/min.

*T*_3_—Measured value of wind loss at 1676 r/min.

The R^2^ of the formulas of 1,381, 1,502 and 1,676 r/min is 95.13, 94.74, and 94.25%. The root mean square error is 0.15, 0.18, and 0.15. The standard error is 0.02, 0.03, and 0.03. The smaller the root mean square error and standard error, the models more stable. According to the metrics above, the BP neural network model can better fit the data.

## Discussion

In this study, the classic regression algorithm, PLSR algorithm, and BP neural network algorithm are used to obtain a wind loss model. The BP neural network model has the highest accuracy, and the classic regression model has the lowest accuracy.

The process of obtaining functional equations through classic regression depends on the selection of equation types. Whether the selection of equation types is appropriate has a great impact on the accuracy of the model. In the process of data regression using classic regression and the PLSR algorithm, the obtained regression equation is more intuitive. The calculation processes of PLSR and classic regression models are highly dependent on the mathematical knowledge of operators. For functional equations with low regression accuracy, it is necessary to carry out stepwise regression. In the BP neural network training process, to obtain a better model and accuracy, it is necessary to set reasonable parameters and approach the objective function through multiple regression training (Lu, 2014). This algorithm cannot directly calculate the regression equation, and it needs a program to calculate the obtained model by importing software (Xin et al., 2002). The BP neural network cannot directly reflect the relationship between the input and output data but can only draw the relationship model of the input and output by tracing points. The BP neural network can be used for the regression of complex models, especially when the relationship of variables in the model cannot be determined. Through the above analysis, it is concluded that the BP neural network regression algorithm is better than the PLSR algorithm and classic regression algorithm. Because the wind loss model obtained by the BP neural network algorithm is not intuitive, PLSR has the advantages of an intuitive and simple model in the three algorithms, which may cause some wind delivery errors in the spraying process. In the process of variable spraying selection, the wind loss model based on a BP neural network or PLSR can be selected according to the required spraying accuracy and spraying error range.

This research shows that there is a correlation between the inner leaf area of the canopy and LiDAR point cloud data, which is consistent with the research results of Sanz-Cortiella et al. (2011); Sanz et al. (2013) and Zhang et al. (2017). However, because these two factors have no significant impact on wind loss, the obtained wind loss model does not present leaf area and LiDAR point cloud data as independent variables in the model. On the one hand, the canopy leaves of orchard trees are not concentrated in the direction of canopy thickness, which has little impact on wind loss; on the other hand, the wind from the sprayer is strong, and the leaves in the canopy have little impact on its loss.

There are many factors affecting the loss of wind power in and out of the canopy. In addition to the thickness of the canopy and the number of leaves in the canopy, the loss is also affected by the growth direction of the branches in the canopy, the leaf inclination of the leaves, and the distribution form of the leaves in the canopy. Measurement methods are also important factors affecting the loss of wind power. In this study, an anemometer is used for multipoint measurement, and the measurement results are more accurate. However, there are shortcomings of low measurement efficiency, and wind measurements in the same measurement area are not obtained at the same time. In future research, multiple anemometers can be used to measure at the same time to reduce the impact caused by anemometer measurements during the testing process.

## Conclusion

In the process of variable wind spraying, the appropriate wind force is determined in real time according to parameters such as canopy size and biomass in the canopy of orchard trees so that the droplets can penetrate the surface of the canopy and deposit into the interior of the canopy for the effective prevention and control of diseases and pests. The influencing factors and models of the wind loss rate in the canopy under different sprayer fan speeds are studied. Through classic regression analysis, the PLSR algorithm, and the BP neural network regression algorithm for data processing and model establishment, the following conclusions are drawn:

Classic regression analysis was used to conduct multiple regression analysis on the relevant factors that were assumed to affect the wind loss rate in the canopy. Under the conditions of different fan speeds of 1,381, 1,502, and 1,676 r/min, the R^2^ values of the obtained model are 0.603, 0.506, and 0.449, respectively. With increasing fan speed, the R^2^ of the obtained canopy wind loss model gradually decreases. The wind force at the entrance of the canopy and the travel of air flow in the canopy are the main factors affecting the wind loss rate. Due to the uneven distribution of leaves in the canopy of orchard trees, the influence of the inner leaf area of the canopy and LiDAR point cloud data on the wind loss rate was not significant and was not studied as an influencing factor. However, it was shown that there was a correlation between the inner leaf area of the canopy and LiDAR point cloud data.

Using the PLSR algorithm and BP neural network algorithm to study the regression model of canopy wind loss can further improve the accuracy of the model. Under the above fan speed conditions, the R^2^ values obtained by the PLSR algorithm are 0.624, 0.653, and 0.569, which are 0.021, 0.147, and 0.120 higher than those of the multiple regression algorithm, respectively. Compared with the above two methods, the BP neural network regression algorithm can significantly improve the fitting accuracy of the model. Under different fan speeds, the determination coefficients R^2^ of the model are 0.783, 0.679, and 0.715, which are 0.18, 0.173, and 0.266 higher than those of the multiple regression analysis.

In this study, combined with the canopy volume and canopy leaf area model, a wind loss rate model under different algorithm conditions is proposed, which provides a reference for the wind control of a sprayer.

## Data availability statement

The raw data supporting the conclusions of this article will be made available by the authors, without undue reservation.

## Author contributions

CG and CZ: conceptualization, validation, investigation, and methodology. CG: software and visualization. CG, LC, and WZ: formal analysis. CG, CZ, and WZ: data curation. WZ and CZ: resources and supervision. CG and WZ: writing—original draft preparation. CG, CZ, LC, WZ, and XW writing—review and editing and funding acquisition. CZ: project administration. All authors contributed to the article and approved the submitted version.

## Funding

This work was financially supported by the Beijing Rural Revitalization Project (grant number: 2022), the National Key Research and Development Plan Project, Robotic Systems for Agriculture (RS-Agri) (grant number: 2019YFE0125200), the Outstanding Scientist Program of Beijing Academy of Agriculture and Forestry Sciences (grant number: jkzx202212), and the Natural Science Foundation of China (grant number: NSFC31971775).

## Conflict of interest

The authors declare that the research was conducted in the absence of any commercial or financial relationships that could be construed as a potential conflict of interest.

## Publisher’s note

All claims expressed in this article are solely those of the authors and do not necessarily represent those of their affiliated organizations, or those of the publisher, the editors and the reviewers. Any product that may be evaluated in this article, or claim that may be made by its manufacturer, is not guaranteed or endorsed by the publisher.
